# Real-world datasets for portfolio selection and solutions of some stochastic dominance portfolio models

**DOI:** 10.1016/j.dib.2016.06.031

**Published:** 2016-06-28

**Authors:** Renato Bruni, Francesco Cesarone, Andrea Scozzari, Fabio Tardella

**Affiliations:** a Dip. Di Ingegneria Informatica, Automatica e Gestionale, Sapienza Università Di Roma, Rome, Italy; b Dip. di Studi Aziendali, Università di Roma Tre, Rome, Italy; c Facoltà di Economia, Università degli Studi Niccolò Cusano, Rome, Italy; d Dip. Metodi e Modelli per l׳Economia, il Territorio e la Finanza, Sapienza Università di Roma, Rome, Italy

## Abstract

A large number of portfolio selection models have appeared in the literature since the pioneering work of Markowitz. However, even when computational and empirical results are described, they are often hard to replicate and compare due to the unavailability of the datasets used in the experiments.

We provide here several datasets for portfolio selection generated using real-world price values from several major stock markets. The datasets contain weekly return values, adjusted for dividends and for stock splits, which are cleaned from errors as much as possible. The datasets are available in different formats, and can be used as benchmarks for testing the performances of portfolio selection models and for comparing the efficiency of the algorithms used to solve them. We also provide, for these datasets, the portfolios obtained by several selection strategies based on Stochastic Dominance models (see “On Exact and Approximate Stochastic Dominance Strategies for Portfolio Selection” (Bruni et al. [2])). We believe that testing portfolio models on publicly available datasets greatly simplifies the comparison of the different portfolio selection strategies.

**Specifications Table**

**Table:** 

Subject area	*Economics and Finance*
More specific subject area	*Portfolio selection, Portfolio optimization, Asset allocation*
Type of data	*Tables, text files, excel files, matlab files, figures*
How data was acquired	*Thomson Reuters Datastream, Fama & French Data Library*
Data format	*Processed, filtered, analyzed*
Experimental factors	*When necessary, the assets prices are filtered to check and to correct missing or inaccurate data*
Experimental features	*All data sets provided consist of weekly assets returns readily usable in Portfolio Selection models*
Data source location	*N/A*
Data accessibility	*Data is within this article*

**Value of the data**

• The datasets provided here can be used as benchmarks by researchers willing to implement and to compare portfolio selection models on publicly available data.
• If different researchers use the same publicly available data, the comparison of different approaches would be more easy and fair.
• The data are filtered to remove possible errors in the original source. This allows researchers to perform more accurate and realistic simulations and evaluations.
• For our datasets we also provide the solutions to several portfolio selection models. Such solutions can be used by other researchers to compare the efficiency of their algorithms and the quality of their solutions.
• Availability of data and solutions can stimulate contacts among researchers working in this area for future collaborations and projects.

## 1 Data

We provide weekly returns time series for assets and indexes belonging to several major stock markets across the world. Weekly returns data are computed from prices values obtained from *Thomson Reuters Datastream* (http://financial.thomsonreuters.com/) and from daily returns obtained from *Fama & French Data Library* (http://mba.tuck.dartmouth.edu/pages/faculty/ken.french/data_library.html). The data are filtered to check and to correct missing or inaccurate values. The data provided can be used as input for several types of portfolio selection models to compare on both efficiency and performance (for references on portfolio selection approaches see, e.g., [3]). For the above datasets, we also include as benchmarks the portfolios obtained by using several selection strategies based on both exact and approximate Stochastic Dominance models (described in [2]).

## 2 Experimental design, materials and methods

Asset allocation aims at selecting a portfolio over N available assets in an investment universe A={1,…,N} according to specific choice criteria under uncertainty. More precisely, we must decide how much of each asset i∈Ashould be purchased in the selected portfolio. The portfolio is denoted by $x=\{x_{1},\ldots ,x_{N}\}$, where $x_{i}$ is the fraction of the given capital invested in asset i∈A.

Let $p_{i,t}$ denote the price of asset i at time t, observed for *m*+1 time periods, i.e., t∈{0,1,…,m}. The linear return of asset i at time t is

$$r_{i,t}=\left(p_{i,t}-p_{i,t-1}\right)/p_{i,t-1}$$

where t∈T={1,…,m}. Denoting by $b_{t}$ the value of the benchmark (e.g., the Market Index) at time t∈{0,1,…,m}, the benchmark linear returns are

$$r_{t}^{I}=\left(b_{t}-b_{t-1}\right)/b_{t-1}$$

where t∈T={1,…,m}. The portfolio linear return at time t∈T is

$$R_{t}(x)=\sum_{i\in A}x_{i}r_{i,t}$$

All the datasets listed in the following Table 1 contain |T|=m linear return values for each of the *N* assets contained in the market, together with the linear returns of the benchmark index, computed as described above.

Datasets 1–5 consist of weekly linear returns computed on daily price data, adjusted for dividends and stock splits, obtained from *Thomson Reuters Datastream*. The selected benchmark is the market index. Stocks with less than ten years of observations were disregarded, thus obtaining a reasonable tradeoff between the number of assets (*N*) and of observations (|*T*|). Furthermore, when necessary, the assets prices are filtered to check and to correct inaccurate data. Data cleaning is indeed an important issue for similar data (see, e.g., [4] for references on this widespread problem).

Dataset 6 is derived from the Fama and French 49 Industry portfolios, available from the Fama & French Data Library, which contains daily returns from July 1926 to July 2015. Since there are many data missing, especially before July 1969, we choose a subsample of H=11628 periods where all the daily returns of the 49 industries are available, namely from July 1969 to July 2015. Furthermore, to standardize the frequencies of all data sets we extract weekly returns $r_{k}^{w}$ by cumulating daily returns $r_{i}^{d}$ in groups of five as follows:

$$r_{k}^{w}=\prod_{j=1}^{5}\left(1+r_{5k+j}^{d}\right)-1,k=0,\ldots ,\left\lfloor \frac{H}{5}\right\rfloor -1.$$

Since no market index is publicly available for the Fama and French 49 Industry portfolios, in this case we use the Equally-Weighted portfolio as a benchmark index.

In addition to the returns datasets, we also make available the composition (weights) and the out-of-sample returns of the portfolios obtained, for all datasets and for several in-sample periods, with the models listed in Table 2 and fully described in the companion paper [2].

**Fig. 1:**
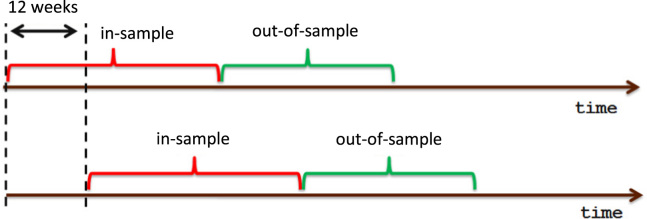
Scheme of the rolling time window used in the analysis.

For each dataset and for each model, we compute the solutions using a rolling in-sample window of 52 returns observations. We initially set the in-sample window on the first 52 time periods, we select the portfolio by solving the model, and we evaluate the performance of the selected portfolio on the following 12 (out-of-sample) periods. Next, we update the in-sample window, with the inclusion of the previous 12 out-of-sample periods and the exclusion of the first 12 periods of the previous in-sample window. We then rebalance the portfolio by solving the model again, and repeat until the end of the dataset (see Fig. 1).

**Fig. 2:**
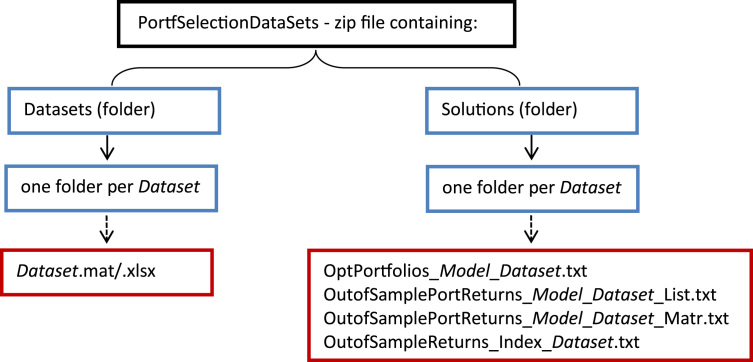
Structure of the database.

Following the notation of Table 1, the data provided with this article are organized as in Fig. 2 and labeled as follows:

• *Dataset.*mat: matlab workspace containing the |T|X *N* returns matrix (*Assets_Returns*) and the |T|X 1 vector of Index returns (*Index_Returns*) for the *Dataset*.
• *Dataset.*xlsx: excel file containing the |T|X *N* returns matrix in the sheet *Assets_Returns* and the |T| X 1 vector of Index returns in the sheet *Index_Returns* for the *Dataset*.
• OptPortfolios_*Model*_*Dataset.*txt: matrix (with size *N* X *nreb*) of portfolio weights obtained by the *Model* for the *Dataset*.
• OutofSamplePortReturns_*Model*_*Dataset*_List.txt: vector (with size |T|-52 X 1) of the out-of-sample portfolio returns obtained by the *Model* for the *Dataset*.
• OutofSamplePortReturns_*Model*_*Dataset*_Matr.txt: same as above but in matlab matrix format.
• OutofSampleReturns_Index_*Dataset.*txt: vector (with size |T|-52 X 1) of the out-of-sample benchmark Index returns for the *Dataset*.

## Figures and Tables

**Table 1:** Weekly returns datasets provided.

	**Dataset Name**	**# of assets (*****N*****)**	|T|	**Time interval**	**Country**	**Description**	**# of rebalancing (*****nreb*****)**
**1**	DowJones	28	1363	Feb 1990-Apr 2016	USA	Dow Jones Industrial Average	110
**2**	NASDAQ100	82	596	Nov 2004-Apr 2016	USA	NASDAQ 100	46
**3**	FTSE100	83	717	Jul 2002-Apr 2016	UK	FTSE 100	56
**4**	SP500	442	595	Nov 2004-Apr 2016	USA	S&P 500	46
**5**	NASDAQComp	1203	685	Feb 2003-Apr 2016	USA	NASDAQ Composite	53
**6**	FF49Industries	49	2325	Jul 1969-Jul 2015	USA	Fama and French 49 Industry	190

**Table 2:** Portfolio Selection models applied to the datasets.

**Model Name**	**Description**
CZeSD	Cumulative Zero-order epsilon Stochastic Dominance (see [1], [2])
RMZ_SSD	Roman-Mitra-Zviarovich Second-Order Stochastic Dominance (see [9])
LR_ASSD	Lizyayev-Ruszczynski approximate Second-Order Stochastic Dominance (see [5])
L_SSD	Luedtke Second-Order Stochastic Dominance (see [6])
KP_SSD	Post-Kopa Second-Order Stochastic Dominance (see [8])
MeanVar	Markowitz Mean-Variance (see [7])
